# Genome-wide studies reveal novel and distinct biological pathways regulated by SIN3 isoforms

**DOI:** 10.1186/s12864-016-2428-5

**Published:** 2016-02-13

**Authors:** Nirmalya Saha, Mengying Liu, Ambikai Gajan, Lori A. Pile

**Affiliations:** Department of Biological Sciences, Wayne State University, Detroit, MI USA

**Keywords:** SIN3 isoforms, *Drosophila melanogaster*, Gene regulation, Histone modification

## Abstract

**Background:**

The multisubunit SIN3 complex is a global transcriptional regulator. In *Drosophila*, a single *Sin3A* gene encodes different isoforms of SIN3, of which SIN3 187 and SIN3 220 are the major isoforms. Previous studies have demonstrated functional non-redundancy of SIN3 isoforms. The role of SIN3 isoforms in regulating distinct biological processes, however, is not well characterized.

**Results:**

We established a *Drosophila* S2 cell culture model system in which cells predominantly express either SIN3 187 or SIN3 220. To identify genomic targets of SIN3 isoforms, we performed chromatin immunoprecipitation followed by deep sequencing. Our data demonstrate that upon overexpression of SIN3 187, the level of SIN3 220 decreased and the large majority of genomic sites bound by SIN3 220 were instead bound by SIN3 187. We used RNA-seq to identify genes regulated by the expression of one isoform or the other. In S2 cells, which predominantly express SIN3 220, we found that SIN3 220 directly regulates genes involved in metabolism and cell proliferation. We also determined that SIN3 187 regulates a unique set of genes and likely modulates expression of many genes also regulated by SIN3 220. Interestingly, biological pathways enriched for genes specifically regulated by SIN3 187 strongly suggest that this isoform plays an important role during the transition from the embryonic to the larval stage of development.

**Conclusion:**

These data establish the role of SIN3 isoforms in regulating distinct biological processes. This study substantially contributes to our understanding of the complexity of gene regulation by SIN3.

**Electronic supplementary material:**

The online version of this article (doi:10.1186/s12864-016-2428-5) contains supplementary material, which is available to authorized users.

## Background

Nucleosomes are comprised of DNA wrapped around histone proteins to form a stable chromatin structure. Post translational modifications (PTMs) of histones influence chromatin structure and the transcriptional state of genes [1]. Histone acetylation, which is one of the earliest discovered histone PTMs [2, 3], has been well established as a transcriptional activation mark [4, 5]. The reverse, histone deacetylation, is correlated with transcription repression [5]. Histone lysine acetyltransferases (KATs) and histone deacetylases (HDACs) are key effectors conferring histone acetylation and deacetylation, respectively. These enzymes are often found in complexes where they associate with a scaffold protein and accessory factors. The accessory proteins are thought to finely tune the enzymatic activity of complexes [6]. One such multiprotein complex is the SIN3/RPD3 HDAC complex, in which SIN3 acts as the master transcriptional adapter protein that interacts with the deacetylase RPD3 and other accessory proteins [7, 8]. The primary role of the SIN3 complex is to mediate gene repression, however, evidence of transcriptional activation promoted by SIN3 has been documented [9–14].

SIN3 activity is linked to several cellular processes throughout metazoan development. SIN3 has been shown to be involved in protein stability, oncogenic transformations, senescence and cell survival [15]. SIN3 is also implicated in cell cycle regulation. Reduction of SIN3 in *Drosophila* S2 cells compromises the G2/M phase transition during cell cycle progression [16]. Additionally, RNA interference (RNAi) mediated knockdown of *Sin3A* causes curved wings in adult flies [17]. Rescue of the curved wing phenotype by overexpression of String, a G2/M regulator, highlights the importance of SIN3 in cell cycle progression [17]. In mouse, knockdown of *mSin3A* impacts both G1 and G2/M phases of the cell cycle [10, 18]. Null mutants of *sin3* in budding yeast show an accumulation of asynchronous cell population in the G2 phase [19]. SIN3 has also been linked to metabolism. Several metabolic processes including glucose metabolism, oxidative metabolism, oxidative phosphorylation, mitochondrial biogenesis, fatty acid oxidation as well as mitochondrial and cellular protein synthesis have been previously reported to be regulated by SIN3 as determined by expression profiling of S2 and Kc *Drosophila* cell lines in which *Sin3A* was knocked down [12]. In addition, studies in yeast and fly models indicate that SIN3 plays a critical role in regulating mitochondrial activity and oxidative stress [20, 21]. In mouse, two genes encode highly related Sin3 proteins, mSin3A and mSin3B [8, 22]*.* Several investigators have shown that the two highly similar mouse Sin3 proteins play distinct roles during development. For example, *mSin3A* is essential for early embryonic development, survival and growth of cultured cells [10, 18], while *mSin3B* plays a regulatory role during the late gestation period of embryogenesis, suggesting the functional non-redundancy of the *mSIN3* genes [23]. Despite being an area of active research, the complete array of functions and complexity of SIN3 regulatory networks remains largely unknown.

The intricate mechanism of gene regulation by SIN3 is augmented by the presence of distinct SIN3 isoforms or discrete SIN3 HDAC complexes in multiple eukaryotic model systems [8]. In *Drosophila,* splicing of a single gene results in the expression of multiple isoforms of SIN3 [24]. Additionally, subcomplexes of SIN3 with and without RPD3 have been isolated [25]. Unlike *Drosophila*, *Saccharomyces cerevisiae* express a single Sin3 protein [26]. Although a single protein is present, multiple complexes containing Sin3 and Rpd3 have been identified, suggesting a functional diversity of yeast SIN3 HDAC corepressor complexes. In budding yeast, Rpd3 predominantly forms a large complex (Rpd3L) and a small complex (Rpd3S) [27–31]. The core components of these complexes are Sin3, Rpd3 and Ume1. Interestingly, Rpd3L acts as a corepressor at promoters of transcribed genes, while Rpd3S suppresses cryptic transcription by recognizing Set2 methylated histones in the gene body [27]. Furthermore, several subcomplexes of mSin3A and mSin3B have been reported [32, 33]. The distinct activities of the different SIN3 complexes are an area of active research. Taken together, these data confirm the presence of functional variations among SIN3 HDAC complexes in different model organisms.

Previously, we demonstrated the existence of distinct SIN3 HDAC complexes in *Drosophila* [34]*.* SIN3 187 and SIN3 220 are the most prevalent isoforms of SIN3 expressed during fly development [35]. SIN3 220 is the predominantly expressed isoform in proliferative cells such as cultured *Drosophila* S2 cells and larval imaginal disc cells, whereas SIN3 187 is the prevalent isoform during the latter stages of embryonic development and in adults [35]. Additionally, expression of the SIN3 220 isoform was found to rescue lethality due to a genetic null allele, while SIN3 187 was essentially unable to suppress the lethal phenotype [34]. Differential spatial and temporal patterns of expression of SIN3 isoforms support the idea that they have distinct functions. Recently, SIN3 187, but not SIN3 220, was found to play an active regulatory role in the mesoderm [36], which is in accord with the expression of SIN3 187 in differentiated cells. Taken together, results from our laboratory and others strongly suggest non-overlapping activity of SIN3 isoforms in *Drosophila*.

Genome-wide binding sites of SIN3 have been previously published by several groups [14, 37, 38], however, no distinction between isoform specific genomic localization was indicated in those studies. To explore the functional differences of SIN3 isoforms in modulating biological processes, we carried out genome-wide assays in *Drosophila* cultured cells that predominantly express one or the other major isoform. To our knowledge, it is the first time the binding sites of SIN3 isoforms across the *Drosophila* genome have been mapped. Interestingly, we found that overexpression of SIN3 187 led to replacement of SIN3 220 at a majority of the genomic sites, indicating that the binding sites of the SIN3 isoforms overlap with each other. RNA-seq analysis, however, demonstrate that SIN3 187 plays unique gene regulatory roles, in addition to having some functions in common with SIN3 220.

## Results

**Fig. 1:**
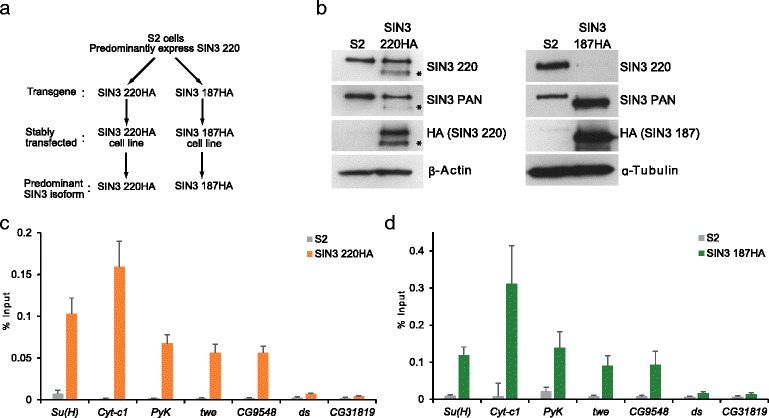
Expression of SIN3 187 affects levels of SIN3 220. **a** Schematic representing the different cell lines used in this study as well as the SIN3 isoform expressed in that line. **b** Western blot analysis of whole cell extract prepared from S2 cells, SIN3 220HA (left) and SIN3 187HA (right) cell lines. The expression of SIN3 220 with a C-terminal HA tag was driven by an inducible metallothionein promoter. Due to leakiness of the metallothionein promoter, the maximum level of expression of SIN3 220HA was achieved without induction. The SIN3 187HA cell line was treated with 0.07 M of CuSO_4_ to induce the transgene. Blots were probed with antibodies listed at the right. SIN3 PAN antibody recognizes all SIN3 isoforms. * denotes degradation product of SIN3 220HA. β-Actin or α-Tubulin was used as a loading control. **c**, **d** ChIP was performed on chromatin prepared from S2 (control), SIN3 220HA (**c**) or SIN3 187HA (**d**) cells using antibody against the HA tag. Immunoprecipitated DNA was quantified by quantitative PCR (qPCR). PCR amplification of ChIP DNA was carried out using primers designed within 500 bp upstream or downstream of the transcription start site (TSS) of all genes. Enrichment of SIN3 isoforms at gene targets is represented as a mean value of the percent of input ± standard error of the mean from three independent biological replicates

To analyze gene regulatory activity of SIN3 isoforms*,* we established two S2 cultured cell lines in which cells express either of the two major SIN3 isoforms with a tag for immunoprecipitation (Fig. 1a). The predominantly expressed isoform of SIN3 in S2 cells is SIN3 220 (Fig. 1a and b). To express SIN3 220 with an HA tag, a stable S2 cell line carrying a transgene with SIN3 220HA cDNA was generated [34] (Fig. 1b, left panel). In addition to the stable SIN3 220HA cell line, we also generated a stable cell line for expression of SIN3 187HA (Fig. 1b, right panel) [34]. Interestingly, overexpression of SIN3 187HA resulted in an almost complete reduction of SIN3 220, when compared to S2 cells (Fig. 1b, right panel). These results indicate that the SIN3 187 isoform modulates the expression of SIN3 220. Important for this study, we have established a homogeneous cell culture system in which cells express almost exclusively one isoform or the other.

Utilizing this cell culture system, we first set out to study the binding of SIN3 isoforms at putative gene targets, predicted from previous studies [12, 37, 38]. To confirm the localization of SIN3 220 to specific targets, we prepared chromatin from the SIN3 220HA cell line and performed chromatin immunoprecipitation (ChIP) using antibody against the HA tag followed by quantitative PCR (qPCR). As predicted, ChIP-qPCR data showed the enrichment of SIN3 220 at putative gene targets *Supressor of hairless* (*Su(H)), Cytochrome c1 (Cyt-c1), Pyruvate kinase (Pyk), twine (twe), CG9548* but not at control regions *dachsous (ds)* or *CG31819* (Fig. 1c). Next, we sought to measure the binding of SIN3 187 to the predicted targets. We performed ChIP on chromatin prepared from the SIN3 187HA cell line using antibody against the HA tag. Following induction of SIN3 187HA for 48 hr, we observed considerable enrichment of SIN3 187 at the same targets bound by SIN3 220 (Fig. 1d), suggesting that SIN3 187 is recruited to the same genes as those bound by the SIN3 220 isoform. Chromatin from the non-transfected S2 cell line was used as a non-specific ChIP control. As expected, little SIN3 enrichment at any of the tested targets was observed in these control cells (Fig. 1c and d). We performed an additional ChIP-qPCR experiment using antibody that recognizes both SIN3 isoforms. The results confirm the recruitment of SIN3 187 to SIN3 220 targeted genomic sites (Additional file 1). Based on the western blot analysis, we conclude that expression of SIN3 187 impacts the level of expression of SIN3 220. Additionally, following ectopic expression of SIN3 187, SIN3 220 is replaced by SIN3 187 at the tested genes. Most importantly, the ChIP-qPCR results demonstrate that we were able to effectively immunoprecipitate chromatin fragments bound by SIN3 isoforms. Next, using ChIP followed by deep sequencing, we set out to map the binding sites of SIN3 isoforms across the entire *Drosophila* genome at a high resolution.

### SIN3 isoforms bind to overlapping genomic targets

Although genome-wide SIN3 occupancy has been previously mapped [14, 37, 38], the genes that are differentially bound by SIN3 isoforms have not been determined. In the current study, we performed chromatin immunoprecipitation followed by high-throughput sequencing (ChIP-seq) from chromatin prepared from the stable cell lines that express either of the SIN3 isoforms tagged with HA (Fig. 1). We performed two independent biological replicates of the ChIP-seq experiment, for which we prepared separate sequencing libraries for SIN3 187HA and SIN3 220HA ChIP DNA samples. Additionally, we prepared separate libraries using input DNA samples for each replicate. Following sequencing of the ChIP samples, we used MACS2 to call peaks at an irreproducible discovery rate (IDR) of 0.1 (Additional file 2 and Additional file 3). Further, we retained only those peaks that were three fold or more enriched over the input sample. 4903 and 5810 peaks were called for the SIN3 187 and the SIN3 220 libraries, respectively. A comparison of SIN3 isoform bound peaks in S2 cells with those of the binding sites of SIN3 mapped previously in the *Drosophila* embryos [38] showed a substantial level of correlation (more than 50 % overlap) (Additional file 4). This overlap between the occupancy of the SIN3 isoforms in S2 cells in this study and published SIN3 binding in *Drosophila* embryos suggest that in the whole organism, SIN3 isoforms are likely recruited to many genomic targets by a mechanism similar to that in S2 cells. Additionally, the differences in the binding patterns suggest that there are tissue-specific binding sites for the isoforms.

**Fig. 2:**
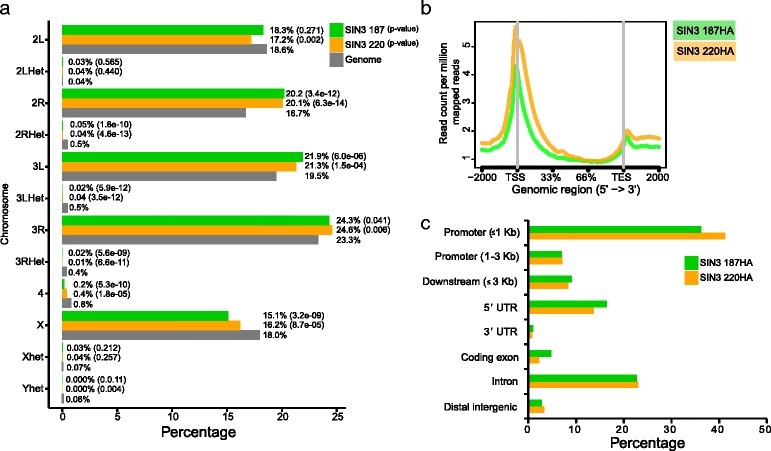
Genome-wide occupancy profile of SIN3 isoforms. **a** The chromosomal distribution of SIN3 isoforms across the *Drosophila melanogaster* genome as determined by the cis-regulatory element annotation system (CEAS). The *P*-value represents the significance of relative enrichment over genome. It was calculated using a one-sided binomial test. **b** The metagene analysis of all peaks showing the enrichment of SIN3 isoforms around the transcription start site (TSS) and the transcription end site (TES). **c** Bar plot representing the enrichment of SIN3 isoforms over genomic features

Through investigation of the chromosomal distribution of SIN3 isoforms, we found that they are enriched over the euchromatic regions of the *Drosophila* genome as determined by the cis-regulatory enrichment annotation system (CEAS) analysis [39]. Specifically, we found that 99 % of the peaks identified for SIN3 isoforms were represented on the euchromatic arms of chromosome 2L, 2R, 3L, 3R and X (Fig. 2a and Additional file 5). This result is in accord with SIN3 binding at euchromatic regions in polytene chromosomes [40]. We found that a large majority of peaks of the SIN3 isoforms localized around the transcription start sites (TSS) of genes, which suggests that both SIN3 isoforms bind the promoter regions of genes to regulate transcriptional activity (Fig. 2b). We also note a slight enrichment of the SIN3 isoforms around the transcription end sites (TES) of genes. We do not rule out the possibility that some of these peaks may overlap with the promoter region of other closely localized genes. The peaks of SIN3 binding were assigned to genomic features and grouped into eight categories (Fig. 2c). More than 50 % of the peaks were located within 1 kb upstream of the TSS and 5’UTR region. Enrichment of SIN3 isoforms beyond 1 kb upstream of the TSS was observed but at a low level. We also note that SIN3 isoforms were preferentially located over introns versus coding exons. Very little or negligible binding of SIN3 isoforms was observed at the 3’UTR or distal intergenic regions.

**Fig. 3:**
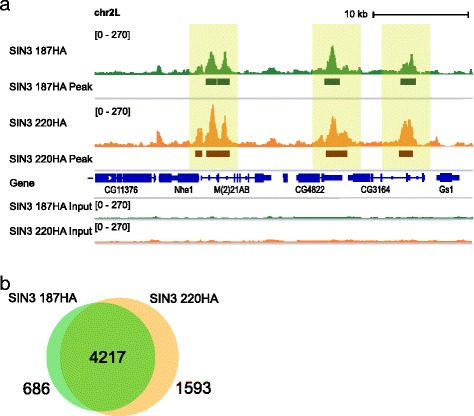
SIN3 isoform binding sites largely overlap. **a** Binding of SIN3 187HA and SIN3 220HA are depicted as standard genomic tracks on the integrated genomic viewer. Peaks were aligned to the built-in *Drosophila melanogaster* gene annotation track, shown in blue. Exons are shown in solid blue, introns as blue lines and arrows indicate the directionality of genes. SIN3 187HA and SIN3 220HA peaks as called by MACS2 are displayed in dark green and dark orange bars, respectively, below their enrichment tracks. Input tracks are shown in green for SIN3 187HA and orange for SIN3 220HA. All peaks shown are highly significant, *P*-value <1e-20. **b** Venn diagram showing the overlap of peaks between the SIN3 187HA and the SIN3 220HA ChIP-seq data. A minimum of 50 % overlap between peaks of the two SIN3 isoforms was considered

The enrichment patterns of SIN3 187 and SIN3 220 at genomic features (promoter, 5’UTR, intron, gene body and 3’UTR) are similar to each other (Fig. 2c). Additionally, a comparison of ChIP-seq profiles of SIN3 isoforms revealed that SIN3 isoforms localize to overlapping genomic loci (Fig. 3a). We next directly compared the peaks identified for SIN3 187 and SIN3 220. We considered the peaks to be bound by SIN3 isoforms as similar only if the sequence of overlap between the peaks was at least 50 %. Using this criterion, we found 86 % of SIN3 187 peaks called overlapped with 73 % of SIN3 220 peaks, indicating that the majority of genomic sites targeted by SIN3 isoforms are common (Fig. 3b). These data demonstrate that expression of SIN3 187 leads to its enrichment at the genomic targets that were previously bound by the SIN3 220 isoform.

Our next goal was to identify genes that are possibly bound by SIN3 isoforms. For this objective we assigned peaks to genes. If a peak under investigation mapped within 1 kb upstream of the TSS and 100 bp downstream of the TES, it was assigned to that gene. Therefore, a sample peak could be assigned to one or more genes, depending on the directionality and the distance between genes. Utilizing this method, we extensively mapped the genes that are bound by SIN3 isoforms. We identified 5903 (approximately 34 % of the *Drosophila* genome) and 6905 (approximately 40 % of the *Drosophila* genome) genes bound by SIN3 187 and SIN3 220, respectively, supporting the idea of a global transcriptional role of SIN3 isoforms. Additionally, the ChIP-qPCR data showing localization of the SIN3 isoforms at putative gene targets (Fig. 1c and d) were validated by the ChIP-seq results (Additional file 6). Taken together, our data demonstrate that SIN3 isoforms are targeted to many overlapping genomic loci and a substantial enrichment of SIN3 is observed around the TSS. Overall, these data are in accord with previous findings showing that SIN3 localizes to euchromatic regions of the genome and the extent of SIN3 binding to the *Drosophila* genome supports previous results indicating that SIN3 is a global transcriptional regulator [37, 38, 40].

### SIN3 220 directly regulates genes involved in metabolism and cell cycle progression

**Fig. 4:**
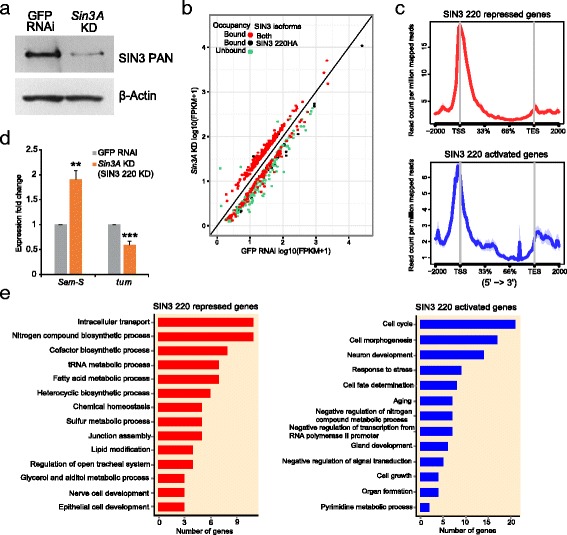
ChIP-seq and transcriptome analysis reveal the genes directly regulated by SIN3 220. **a** Western blot for SIN3 of whole cell extracts prepared from GFP RNAi or *Sin3A* knockdown (KD) cells. β-Actin was used as a loading control. **b** Scatter plot showing the correlation between genes bound by SIN3 220HA and those that change in expression following *Sin3A* knockdown. Green dots denote genes regulated by SIN3 220 but not bound. Red dots indicate genes that are regulated by SIN3 220 and bound by both SIN3 isoforms. Black dots refer to genes uniquely bound and regulated by the SIN3 220 isoform. **c** Average ChIP signal of SIN3 220HA over the genes as identified in (**b**). **d** qRT-PCR verification of genes identified by RNA-seq to change in expression upon *Sin3A* knockdown versus GFP RNAi. *P*-value ** <0.01, *** <0.0001. **e** Gene-ontology analysis of the genes identified in (**b**) are represented as bar plots. The Y-axis lists the broad GO categories. Red: categories of genes that are directly repressed. Blue: categories of genes directly activated. *P*-value (gene ontology categories) <0.05

The high resolution ChIP-seq profile identified genomic targets of the SIN3 220 isoform. To investigate the genes regulated by the SIN3 220 isoform, we performed transcriptome analysis by RNA-seq on total mRNA isolated from S2 cells treated with *Sin3A* dsRNA or GFP dsRNA as a control. As SIN3 220 is the predominant isoform in S2 cells, we considered genes misregulated upon *Sin3A* knockdown in S2 cells to be regulated by the SIN3 220 isoform and we refer to these genes as SIN3 220 targets. Efficient knockdown of *Sin3A* was verified by western blot (Fig. 4a). For the expression profile we selected genes that showed a change in expression of ≥1.5 fold (FDR ≤ 0.05) for *Sin3A* knockdown samples compared to GFP RNAi samples. We identified 602 genes as misregulated upon reduction of *Sin3A* in S2 cells (Additional file 7). 263 were upregulated and 349 genes were downregulated upon *Sin3A* knockdown. Based on this RNA-seq analysis, the SIN3 220 isoform acts as both a co-repressor, for those genes upregulated upon *Sin3A* knockdown, and as a co-activator, for the genes demonstrating a decrease in expression following *Sin3A* knockdown.

To identify the genes regulated directly by SIN3 220, we integrated the SIN3 220 ChIP-seq and the *Sin3A* knockdown RNA-seq data. We retained only those genes that responded to reduced SIN3 expression and were bound by SIN3 220. Approximately 92 % (243/263) and 46 % (162/349) of the genes repressed and activated by SIN3 220, respectively, are direct targets (Fig. 4b, red and black dots). This finding is particularly interesting because the data suggest that genes repressed by SIN3 220 are more likely to be direct targets in comparison to the genes activated by SIN3 220. We next performed metagene analysis on genes directly regulated by SIN3 220 to study the distribution across gene features. A large majority of SIN3 220 peaks were found at the transcription start sites of the repressed and activated genes (Fig. 4c). We also observed some enrichment around the transcription end site, exclusively at genes activated by SIN3 220. We note that the majority of the genes directly regulated by SIN3 220 are also bound by SIN3 187, as indicated by the SIN3 187 ChIP-seq data (Fig. 4b, red dots). Regulation of these genes by SIN3 187 is explained in more detail below.

The genomic experiments identified several genes directly regulated by SIN3 220. To verify the RNA-seq data, we selected genes that showed a change in expression upon *Sin3A* knockdown and were bound by SIN3 220 as determined by the ChIP-seq analysis. We utilized reverse transcription followed by quantitative PCR (RT-qPCR) to verify gene expression changes resulting from *Sin3A* knockdown. We selected *S-adenosyl methionine synthetase* (*Sam-S*) and *tumbleweed* (*tum*), which were found to be altered upon *Sin3A* knockdown, and bound by SIN3 220 as well (Additional file 8). Consistent with the RNA-seq data, we found that *Sam-S* was upregulated upon *Sin3A* knockdown, while expression of *tum* was reduced (Fig. 4d)**.** These data indicate that in S2 cells, SIN3 220 acts as a co-repressor for *Sam-S* and co-activator for *tum.*

Next, we sought to investigate the biological processes that are regulated by SIN3 220. We performed gene ontology (GO) analysis on the genes that were directly repressed and activated by SIN3 220 using the DAVID gene annotation module (Fig. 4e and Additional file 9). 52 % (126/243) of the repressed genes and 57 % (92/162) of the activated genes were enriched for GO terms. Metabolic pathways associated with perturbed levels of SIN3 have been characterized in S2 and Kc cell lines [12]. Consistent with the published data, the category of metabolism was overrepresented in the genes repressed by SIN3 220. In addition to the metabolic and biosynthetic processes, SIN3 220 repressed genes are linked to intracellular transport and junction assembly processes (Fig. 4e). Additionally, GO analysis revealed several well studied and novel biological processes overrepresented by genes activated by SIN3 220 (Fig. 4e). SIN3 220 was found to be important for activation of several genes involved in cell cycle. This is particularly interesting because SIN3 was previously implicated in cell cycle regulation [12]. Data from this current study indicate that the SIN3 220 isoform directly modulated expression of genes involved in cell cycle progression. The GO analysis indicated that SIN3 220 activated genes include those that regulate stress response and aging, which is in accord with our previous findings [21]. Furthermore, these data demonstrate that SIN3 220 regulates genes involved in novel pathways such as cell morphogenesis, cell fate determination and cell growth. Taken together, our findings in S2 cells demonstrate that SIN3 220 plays a critical role in mediating expression of genes encoding proteins involved in a wide array of biological processes. Consistent with the widespread role of histone modifications in modulating gene expression, the gene regulatory transcriptional network controlled by SIN3 220 is quite diverse.

### SIN3 187 regulates many similar genes as those regulated by SIN3 220

**Fig. 5:**
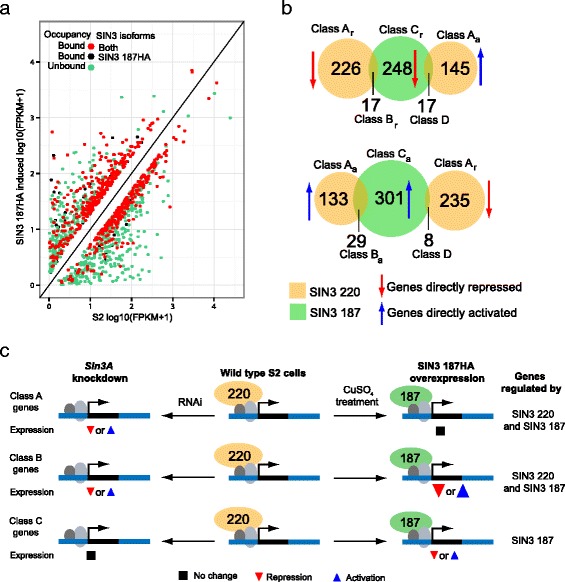
SIN3 187 regulates distinct as well as common genes as that of SIN3 220. **a** Scatter plots showing the correlation between genes bound and that change in expression upon overexpression of SIN3 187. Green dots denote genes regulated by SIN3 187 but not bound. Red dots indicate genes that are regulated by SIN3 187 and are bound by both SIN3 isoforms. Black dots refer to genes uniquely bound and regulated by SIN3 187. **b** Venn diagrams showing the comparison of genes directly regulated by SIN3 isoforms. Different classes of genes are labelled as A, B, C and D. r, repression. a, activation. **c** Schematic showing the regulation of Class A, B and C genes by the SIN3 isoforms. The middle column shows the genes bound by SIN3 220 in wild type cells. Gene expression upon *Sin3A* (SIN3 220) knockdown is depicted in the left column and gene expression upon ectopic expression of SIN3 187HA is represented in the right column

Our findings demonstrate that the ectopic expression of SIN3 187 in S2 cells led to a reduction in the level of SIN3 220 (Fig. 1b). In addition, we found that SIN3 220 was replaced by SIN3 187 at genomic targets (Figs. 1 and 3a). During early *Drosophila* embryogenesis, the levels of SIN3 isoforms are comparable [35]. During the final stage, however, the relative level of the SIN3 220 isoform is reduced with an increase in expression of SIN3 187 [35]. Based on these data, we hypothesized that SIN3 187 likely regulates a different set of genes from those of SIN3 220. To test our hypothesis, we utilized RNA-seq to perform differential gene expression analysis comparing the expression of genes in cells that express SIN3 187HA to the gene expression of non-transfected S2 cells (Fig. 1b, right panel). Overexpression of SIN3 187 leads to the loss of SIN3 220 isoform in S2 cells, thus making this system ideal to investigate the regulatory roles of SIN3 187. Since SIN3 187HA was overexpressed in S2 cells, we used a stringent threshold of ≥2 fold gene expression (FDR ≤ 0.001) alteration between the wild type S2 cells and cells that express SIN3 187HA. Using this criterion, we identified 1274 genes to be differentially regulated due ectopic expression of SIN3 187. The number of genes repressed and activated by SIN3 187 is 669 and 605, respectively (Additional file 7 and Additional file 10). To identify the genes directly regulated by SIN3 187, we combined the binding profile of SIN3 187 with the gene expression changes due to SIN3 187 overexpression. By combining the recruitment data and gene expression changes, we found SIN3 187 bound approximately 42 % (282/669) of repressed genes and 56 % (338/605) of activated genes (Fig. 5a), indicating direct regulation of these genes by SIN3 187.

So far in our analysis, we have identified direct targets of SIN3 187 and SIN3 220 in S2 cells. We next made four comparisons (Fig. 5b): 1. genes directly repressed by SIN3 220 to those directly repressed by SIN3 187, 2. genes directly activated by SIN3 220 to those directly activated by SIN3 187, 3 and 4. genes directly regulated by SIN3 187 and SIN3 220 in opposing directions. Little overlap between gene sets was observed. These data imply that the isoforms are regulating very different sets of genes. Careful consideration of the model system, however, suggests in fact, that this is not the case. These comparisons yielded four classes of genes: A. Genes directly repressed (A_r_) or activated (A_a_) by SIN3 220, B. Genes directly repressed (B_r_) or activated (B_a_) by SIN3 220 as well as SIN3 187, C. Genes directly repressed (C_r_) or activated (C_a_) by SIN3 187 alone and D. Genes that are directly regulated in opposing directions by SIN3 187 and SIN3 220 (Fig. 5b and c). For Class A genes, we did not observe gene expression changes between wild type S2 cells and cells that overexpress SIN3 187. This, however, does not exclude a role for SIN3 187 at Class A genes. Ectopic expression of SIN3 187 replaces SIN3 220 and thus may perform the same role as SIN3 220 without additional expression changes at gene targets compared to wild type cells. 95 % (214/226) and 62 % (83/133) of the directly repressed and directly activated Class A genes, respectively, are bound by SIN3 187, suggesting that majority of these genes are likely regulated by both SIN3 isoforms (Fig. 5c). Class A genes bound only by SIN3 220 are likely to be unique targets of SIN3 220 (Fig. 4b, black dots). Surprisingly, the majority of the genes specifically bound by SIN3 220 were activated. Of note, we cannot rule out the possibility that SIN3 187 might also be recruited to these potential SIN3 220 unique genes, as SIN3 187 peaks might not have been identified in the ChIP-seq assay due to statistical thresholds. A small number of genes were either repressed or activated by both SIN3 187 and SIN3 220 (Class B). All Class B genes are directly regulated by both SIN3 isoforms, suggesting that SIN3 220 and SIN3 187 regulate Class B genes in a similar way. SIN3 187, however, led to increased changes in expression of these genes relative to the wild type cells, implying that SIN3 187 has a stronger effect in regulating these genes compared to SIN3 220 (Fig. 5c). Of high interest are the Class C genes, which are genes uniquely regulated by SIN3 187. Class C genes will be discussed in detail below. Another set of genes falls into Class D, which includes genes that are regulated in opposing directions by SIN3 187 and SIN3 220 (Fig. 5b). Using RT-qPCR, we validated the differential effects of the isoforms on two representative genes of this class (Additional file 11). This class also includes genes that are potentially regulated only by SIN3 220. This gene set may show a change in expression upon overexpression of SIN3 187 due to the loss of SIN3 220 rather than enrichment of SIN3 187. Taken together, we conclude that majority of the genes that are directly regulated by SIN3 220 are also regulated by SIN3 187 in a similar fashion. In addition, we identified several genes to be distinctly regulated by SIN3 187.

### SIN3 187 plays distinct roles to that of SIN3 220 in regulating gene expression

**Fig. 6:**
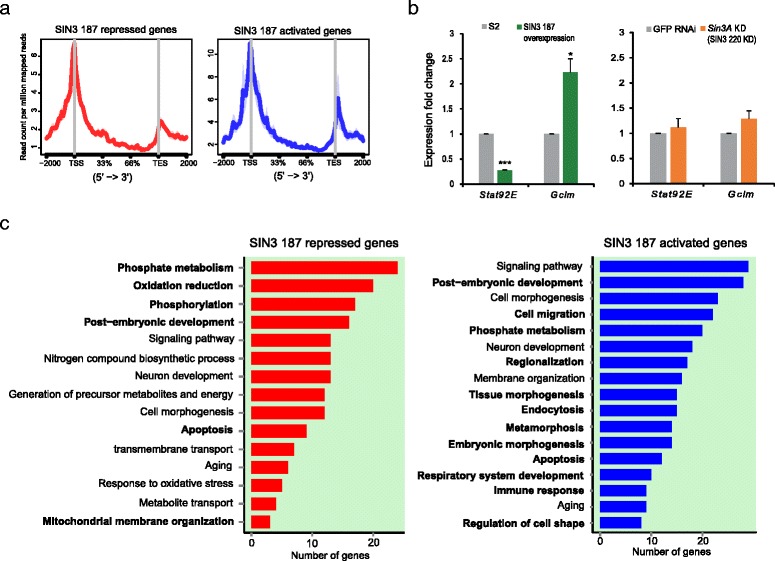
SIN3 187 distinctly regulates differentiation related genes. **a** Average ChIP signal of SIN3 187HA over the genes directly regulated by SIN3 187. **b** qRT-PCR verification of genes identified by RNA-seq to change in expression after overexpression of SIN3 187 versus control (left) but not affected upon *Sin3A* knockdown versus GFP RNAi (right). *P*-value * <0.05, *** <0.0001. **c** Gene ontology analysis of the Class C genes identified in Fig. 5 are represented as bar plots. The Y-axis lists the broad GO categories. Red: categories of genes that are directly repressed. Blue: categories of genes that are directly activated. *P*-value (gene ontology categories) <0.05. Biological processes labelled in bold indicate the unique processes that are overrepresented for genes distinctly regulated by SIN3 187

We performed metagene analysis on genes directly repressed and activated by SIN3 187. This analysis revealed that the preferential binding sites of SIN3 187 are the transcriptional start sites of genes (Fig. 6a). Similar to the observation for SIN3 220 binding, we found substantial enrichment of SIN3 187 around the transcriptional end sites of genes that are activated upon SIN3 187HA overexpression compared to those that are repressed (Fig. 6a). Interestingly, 95 % of the genes directly repressed and 90 % of the genes directly activated by SIN3 187 were genes also bound by the SIN3 220 isoform in the SIN3 220HA cell line (Fig. 5a, red dots). These findings indicate that upon expression of SIN3 187HA, the SIN3 187 isoform occupied the same sites previously bound by SIN3 220. Interestingly, a number of these commonly bound genes demonstrated a change in expression upon ectopic SIN3 187 expression, but not upon *Sin3A,* essentially SIN3 220, knockdown. Thus, SIN3 187 specifically modulates expression of this set of genes (Fig. 5c, Class C). We identified 248 genes repressed and 301 activated uniquely by SIN3 187. Next, we selected two genes to verify the gene regulatory functions of SIN3 187. Based on the RNA-seq data, we chose *Signal-transducer and activator of transcription protein at 92E* (*Stat92E*) and *Glutamate-cysteine ligase modifier subunit* (*Gclm*), which are shown to be repressed and activated specifically by the SIN3 187 isoform, respectively. In addition, these genes are bound by SIN3 187 at the promoter region as determined by the ChIP-seq data (Additional file 8). Using RT-qPCR, we demonstrate that overexpression of SIN3 187 led to repression of *Stat92E* and activation of *Gclm*, thus validating the RNA-seq experiment (Fig. 6b). Furthermore, we analyzed the expression of these genes upon knockdown of *Sin3A* (SIN3 220). Our results demonstrate that reduction of *Sin3A* (SIN3 220) did not impact the expression of these genes (Fig. 6b), although SIN3 220 was bound to those genes in wild type cells (Additional file 8). These results indicate that *Stat92E* and *Gclm* genes are specific targets of SIN3 187 activity.

Our previous research demonstrated that during the late stages of *Drosophila* embryogenesis, expression of SIN3 187 increases and the relative level of the SIN3 220 isoform is reduced [35]. These results led to the hypothesis that SIN3 187 might play a specific role during the latter stages of embryo development. In the current study, we identified the genes specifically regulated by SIN3 187 and bound by SIN3 187 in cultured cells (Fig. 5, Class C). Next, we sought to examine the biological processes that are represented by these genes. We performed GO analysis on the unique direct SIN3 187 targets (Additional file 9). Approximately, 63 % (157/248) of the genes repressed and 56 % (170/301) of the genes activated by SIN3 187 were enriched for GO terms (Fig. 6c). Strikingly, the GO analysis demonstrates that SIN3 187 regulated genes are involved in post-embryonic development, metamorphosis and apoptosis. Interestingly, these categories of biological processes are largely specific to SIN3 187 regulated genes when compared to the genes regulated upon *Sin3A* (SIN3 220) knockdown (compare Figs. 6c and 4e). Additionally, we found that genes involved in phosphate metabolism, phosphorylation, endocytosis, embryonic morphogenesis and development of respiratory system were regulated specifically by the SIN3 187 isoform. Further, we identified a few biological processes regulated by SIN3 187 that were also impacted upon *Sin3A* (SIN3 220) knockdown. For example, biological processes such as neuron development, cell morphogenesis and aging are overrepresented by both SIN3 187 regulated genes and genes that show a change in expression due to reduction of *Sin3A* (SIN3 220). Collectively, these data indicate that SIN3 187 specifically modulates biological processes involved in differentiation and development, in addition to regulating processes in common with the SIN3 220 isoform.

## Discussion

**Fig. 7:**
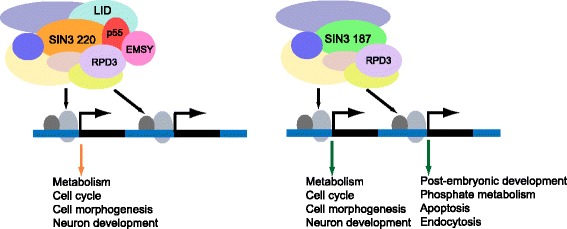
A model depicting the role of SIN3 isoforms in the regulation of gene expression

In this research, we have described the differential role of the SIN3 isoforms in regulating gene activity, summarized in Fig. 7. We have shown that the level of the largest isoform, SIN3 220, is regulated by ectopic expression of SIN3 187. In addition, upon overexpression of SIN3 187, SIN3 187 replaces SIN3 220 at genomic targets. Based on the ChIP-seq data, we determined that SIN3 isoforms localize to overlapping genomic regions, the large majority of which occupy the promoter region of genes. Additionally, global transcriptome analysis revealed that SIN3 isoforms play overlapping roles in regulating processes involved in metabolism, cell cycle and aging. Interestingly, we identified unique genes modulated by SIN3 187. These genes are overrepresented for biological processes that link the activity of SIN3 187 to the control of genes encoding proteins that function in post-embryonic development, endocytosis, phosphate metabolism and embryonic morphogenesis. Together, the current study demonstrates functional overlap as well as non-redundancy of SIN3 isoforms.

In cultured *Drosophila* cells, ectopic expression of SIN3 187 led to a reduced level of SIN3 220 (Fig. 1). This switch in expression mimics the expression pattern of SIN3 isoforms observed during *Drosophila* embryonic development [35]. Together, the fly and S2 cell culture data suggest that SIN3 187 regulates the expression and/or stability of the SIN3 220 isoform. The mechanism through which ectopic expression of SIN3 187 may affect the amount of SIN3 220, however, remains unknown. Preliminary studies from our laboratory suggest that both transcriptional and post-transcriptional regulation is occurring in the cells (data not shown). The ChIP data indicate that ectopically expressed SIN3 187 replaces SIN3 220 at genomic targets. We also determined that the enrichment of SIN3 187 at genomic loci was dependent on the level of SIN3 220 present (data not shown). Therefore, it is plausible that a critical ratio of SIN3 220 to SIN3 187 is essential to determine which of the two isoforms will occupy a gene target, thereby modulating the transcriptional outcome. Additionally, the stage specific expression of SIN3 isoforms during the life cycle of *Drosophila melanogaster* [35] favors the idea that maintenance of a critical ratio of SIN3 isoforms is important.

The gene expression analysis demonstrates that SIN3 187 might play similar roles as that of SIN3 220 as well as carry out unique functions. It is therefore curious that, unlike SIN3 220, expression of SIN3 187 alone does not rescue fly lethality caused by a null mutation in the *Sin3A* gene [34]. One possibility is that SIN3 187 plays a critical gene regulatory role only in the presence of SIN3 220. This scenario is supported by the finding that expression of both SIN3 187 and SIN3 220 in a *Sin3A* null background led to a greater percentage of surviving flies compared to expression of SIN3 220 alone [34]. Additionally, we determined that SIN3 187 is recruited to similar gene targets as that of SIN3 220. We speculate that localization of SIN3 187 to chromatin is dependent or partially dependent on SIN3 220. The preferential interaction of SIN3 220 with p55, LID and EMSY [34] suggests a differential genomic binding capacity of the SIN3 220 isoform that might facilitate the subsequent recruitment of SIN3 187. p55, a WD-40 repeat containing protein, has been demonstrated to provide an interaction framework for histones and other proteins to form multimeric complexes [41–43]. Therefore, it is likely that p55 plays an important role in the chromatin recruitment of SIN3 220. LID facilitates the removal of the histone H3K4 trimethyl mark [44–46]. A recent study using a mouse cell line revealed that the level of histone H3K4me3 influences the recruitment of mSin3A to target sites [47]. Therefore, the LID-containing SIN3 220 complex might alter the chromatin composition in a way that enables subsequent recruitment of SIN3 187.

One of the most surprising findings from our study is that despite the extensive amount of overlapping localization of SIN3 isoforms, the SIN3 187 isoform regulates the level of expression of a distinct set of genes (Fig. 6). One possibility for this result is that binding of SIN3 187 leads to differential chromatin modification. We have previously shown that ectopic expression of SIN3 187 significantly affects both global histone H3K9 and H3K14 acetylation [34]. On the other hand, global changes of only histone H3K9 acetylation were observed upon overexpression of SIN3 220 [34]. In addition, the SIN3 187 isoform specific complex might recruit other *trans*-factors to chromatin, thereby altering transcription in distinct ways. To identify the mechanisms by which SIN3 isoforms regulate gene expression, more extensive evaluation of the interplay of *cis*-elements and *trans*-factors in relation to the SIN3 complexes is required.

Although we mapped the occupancy of SIN3 isoforms to approximately 6000 genes, the transcriptome analysis in S2 cells showed that expression of approximately 1000 genes is affected due to alteration in the level of SIN3 isoforms. We question why many binding events of the SIN3 isoforms do not affect gene expression (Figs. 3, 4 and 6). One explanation is that S2 cells do not represent the complexity of gene regulation in the whole organism. SIN3 isoforms might contribute to gene expression changes depending on their interaction with stage specific transcription factors during the developmental stages of the whole organism. In addition, we do not rule out the possibility that some fortuitous associations of SIN3 isoforms occur, which do not contribute to gene expression alterations, a possibility that has been previously suggested [48, 49]. A recent publication reported a list of putative false positive binding peaks referred to as phantom peaks [50]. The authors suggested that these regions are occupied by any transcription factor irrespective of their biological relevance. Approximately 40 % of peaks identified for SIN3 isoforms by our ChIP-seq assay overlapped with the phantom peaks (data not shown). Approximately 10 % of these peaks, however, can be assigned to genes that demonstrated expression changes due to alterations in the level of SIN3 isoforms, indicating that enrichment of SIN3 isoforms at these loci have functional outcomes.

The large majority of peaks identified for the SIN3 isoforms, mapping around the 5’ end of genes (Figs. 2 and 6), is consistent with observations in other model systems. Interestingly, we observed a substantial level of enrichment of the SIN3 isoforms around the transcription end sites of the genes activated by SIN3 isoforms (Figs. 4 and 6). Alteration of gene expression due to changes in higher order chromatin structure has been reported and reviewed [51]. Therefore, we wonder if there is any possibility that gene looping influences the function of SIN3 isoforms as co-activators of genes. More extensive studies, however, are required to understand the possibility of gene looping and its role in gene regulation by SIN3 isoforms.

## Conclusion

In summary, our results indicate complex mechanisms through which genes are regulated by the SIN3 187 and 220 isoforms. This result suggests that the highly similar SIN3 isoforms likely work in concert with other factors that differentially affect transcript levels. These data suggest a novel mechanism of gene regulation by SIN3 isoforms, by which the SIN3 isoforms localize to overlapping genomic sites and modulate some similar and many distinct biological processes. Collectively, these findings set a solid framework for further understanding of the network of biological processes regulated by SIN3 histone modifying complexes.

## Methods

### Cell culture

The *Drosophila* S2 cells were cultured in *Drosophila* Schneider’s media (1X) + L-glutamine supplemented with 10 % heat-inactivated fetal bovine serum (Invitrogen). Gentamycin (50 mg/ml) was added to S2 cells. S2 cell lines stably transfected with expression constructs of HA-tagged SIN3 187 and SIN3 220 were grown in the media with 0.1 mg/ml of penicillin/streptomycin and 0.1 mg/ml Geneticin. Cells were maintained at 27 °C.

### Western blot

Western blot was carried out following standard protocols [52]. To prepare whole cell extract, approximately 1.5x10^6^ cells were harvested by centrifugation and lysed in Laemmli Sample buffer (Bio-Rad). Proteins (15 to 20 μg) were resolved on an 8 % SDS-polyacrylamide gel and transferred to a polyvinylidine difluoride (PDVF) membrane (Pall). Membranes were incubated with primary antibody: HA-HRP (1:6000; Sigma), SIN3 PAN (1:2000 [40]), SIN3 220 (1:2000 [35]), α-Tubulin (1:1000, Cell signaling) or β-Actin (1:1000, Cell signaling). Following incubation with primary antibody, membranes were incubated with donkey anti-rabbit HRP-conjugated IgG (1:3000, GE healthcare) secondary antibody wherever applicable. The antibody signal was detected using the ECL prime western blot detection system (GE Healthcare).

### Chromatin preparation by MNase digestion

Approximately 4x10^7^ exponentially growing S2 cells were subject to cross-linking by the addition of formaldehyde to a final concentration of 1 % for 10 min at room temperature. Excess formaldehyde was quenched by addition of glycine to a final concentration of 125 mM. The cross-linked cells were washed with 1X phosphate buffered saline and resuspended in lysis buffer (10 mM Tris-HCl pH 8, 10 mM KCl, 3 mM CaCl_2_, 0.34 M sucrose, 1 mM DTT, 0.1 % Triton X-100, 0.2 mM EGTA, proteinase inhibitor (Roche)), followed by incubation on ice for 15 min. These cells were disrupted by dounce homogenizer (10 times by loose pestle, 15 times by tight pestle) followed by low speed centrifugation (170 x g for 10 min). The pellet was resuspended in 200 μl MNase digest buffer (15 mM Tris-HCl pH 8, 60 mM KCl, 15 mM NaCl, 1 mM DTT, 0.25 M sucrose, 1 mM CaCl_2_) and subjected to micrococcal nuclease digestion by addition of 20 units of MNase (Worthington Biochemicals) for 10 min at room temperature. 10 mM EDTA was added to quench the MNase activity. Following MNase digestion, samples were made up to 1.2 ml with resuspension buffer (140 mM NaCl, 10 mM Tris-HCl pH 7.6, 2 mM EDTA) and sonicated using a probe sonicator (Fisher Scientific) to extract cross-linked chromatin in the solution.

Approximately 75 μg of crude chromatin was used to prepare input for the ChIP experiment. Briefly, samples were treated with 0.05 μg/μl of RNase A (Sigma) and incubated overnight with 200 mM NaCl at 65 °C. Samples were treated with 0.04 μM Proteinase K (Fisher Scientific), 10 μM EDTA and 20 μM Tris (pH 8) and incubated at 45 °C for 1.5 h. DNA was purified by standard phenol chloroform extraction. This method leads to the formation of mono, di, tri-nucleosomal DNA fragments as determined by agarose gel electrophoresis analysis of purified DNA.

### Chromatin immunoprecipitation

For HA-tag ChIP, we used monoclonal anti-HA-agarose antibody (Sigma). Anti-HA-agarose bead slurry was washed with RIPA buffer. 75 μg of chromatin was incubated with 30 μl washed anti-HA-agarose for 4 h at 4 °C. Chromatin prepared from wild type S2 cells was used as control. Chromatin bound HA-agarose was then washed, once with 1 ml of modified 2X RIPA buffer (50 mM Tris pH 7.6, 280 mM NaCl, 2 mM EDTA, 0.3 % SDS) for 8 min at room temperature, once with 1 ml of high salt buffer (25 mM Tris pH 7.6, 500 mM NaCl, 1 mM EDTA, 0.1 % SDS, 1 % Triton X-100) at 4 °C for 10 min, once with 1 ml of low salt buffer (10 mM Tris pH 7.6, 250 mM LiCl, 1 mM EDTA, 0.5 % sodium deoxycholate, 0.5 % Triton X-100) at 4 °C for 10 min and once with 1 ml of Tris-EDTA (pH 8). To perform SIN3 PAN ChIP, we incubated chromatin with 10 μl polyclonal SIN3 antibody [40] overnight at 4 °C. Chromatin incubated with pre-immune IgG was used as the non-specific ChIP control. We added 30 μl of washed Protein-A-agarose beads to the anti-SIN3 or pre-immune IgG incubated chromatin followed by incubation for 4 h at 4 °C. Bound beads were washed once with 1 ml of 1x RIPA at 4 °C for 10 min, once with 1 ml of high salt buffer at 4 °C for 10 min, once with 1 ml of low salt buffer at 4 °C for 10 min and once with 1 ml of TE buffer. For both the HA-tag and the anti-SIN3 ChIP, cross-linked protein-DNA was eluted in elution buffer (1 % SDS, 0.1 M NaHCO_3_) at 65 °C for 30 min. Elution was done twice in a total volume of 500 μl. Cross-links were reversed in the eluted samples as for the ChIP input DNA preparation. Purified DNA samples from HA-tag ChIP were quantified by real-time PCR using 2^-ΔΔCT^ method of analysis [53]. All genes tested by ChIP-qPCR validate the ChIP-seq results (Additional file 6). All validation experiments were performed at least three times and data are represented as the mean of percentage of input. Information about the primers used for ChIP-qPCR assay is reported in Additional file 12. HA-tag ChIP was used for the ChIP-seq experiment.

### ChIP-seq library preparation

We prepared sequencing libraries using the method previously described by Ford et al., 2014 [54]. In brief, we used approximately 10 ng of ChIP or input samples to prepare sequencing libraries. ChIP or input DNA was end repaired, adenylated and ligated to indexed adapters. Gel extraction of ligated adapter DNA fragments was carried out on a 2 % low-melting agarose gel (Lonza). 5 cycles of PCR was conducted to convert all Y-shaped structure adapter-DNA fragments to double-stranded DNA structure as previously described [54]. This step was followed by size selection of DNA samples by gel extraction. Finally, size selected DNA fragments were amplified for 12 cycles by PCR. Together, we used 17 cycles of PCR to amplify adapter ligated ChIP DNA. In all steps, DNA was purified by Beckman Coulter Ampure XP resin. The prepared sequencing libraries were sent to the University of Wisconsin Biotechnology Center, where all sequencing samples were multiplexed and sequenced on an Illumina HiSeq2500 platform sequencing system in single end mode with a read length of 100 bp. Two independent biological replicates of the ChIP-seq experiment were performed. ChIP-seq data are available through GEO under GSE72173.

### ChIP-seq analysis

To map the binding sites of SIN3 isoforms, we used Bowtie2 [55] to align all reads to the *Drosophila* genome. Reference *Drosophila melanogaster* sequence (dm3) was downloaded from the UCSC table browser and raw reads were aligned to the reference genome. Only uniquely mapped tags were subsequently used for further analysis. Input DNA tags were used to normalize ChIP DNA tags. Protein bound regions were called using Model-based analysis of ChIP-seq (Macs 2.1.0) [56] using the default parameters and --keep-dup=all. The correlation of the two biological replicates was performed through analysis of the Irreproducible Discovery Rate (IDR) [57–59](Additional file 2 and Additional file 3). Final peaks were called using an IDR of 0.1 %.

Peaks were assigned to UCSC Refseq genes (dm3). Using the bedtools [60] windows function, we assigned peaks within−1 Kb of transcription start sites (promoter) to +100 bp of transcription end sites to genes. We applied the ngsplot tool [61] to perform the metagene analysis and represented the data as the average ChIP signal from−2 Kb of the TSS to + 2 Kb of the TES. Venn diagrams were plotted in R (version: 3.1.1).

### RNA interference

RNAi mediated *Sin3A* knockdown was carried out based on a published protocol [16] with slight modifications. In brief, approximately 4 x 10^6^ cells were plated in 4 ml of *Drosophila* Schneider’s medium. After 3 h, FBS-containing media was carefully removed and supplemented with 2 ml of serum free media. 50 μg of dsRNA was added to plates and mixed by swirling. After 30 min, 4 ml of complete *Drosophila* media was added. Cells were incubated for 4 days. Construction of dsRNA targeting *Sin3A* was previously described [16]. dsRNA against GFP was used as a control. dsRNA targeting GFP was generated using T7 polymerase mediated in vitro transcription of a PCR DNA template (kindly provided by Dr. Russell L. Finley, Jr.) coding *GFP* sequence. The primer pairs to amplify *GFP* containing sequence are GAA TTA ATA CGA CTC ACT ATA GGG AGA TGC CAT CTT CCT TGA AGT CA (forward primer) and GAA TTA ATA CGA CTC ACT ATA GGG AGA TGA TGT TAA CGG CCA CAA GTT (reverse primer). Efficient knockdown of *Sin3A* compared to GFP RNAi was verified by western blot analysis.

### Overexpression of SIN3 187HA

S2 cells carrying stably transfected HA-tagged SIN3 187 transgene under the metallothionein promoter was induced by addition of 0.07 M CuSO_4_. Ectopic expression of SIN3 187HA was verified by western blotting.

### Gene expression analysis by RT-qPCR

Gene expression analysis by RT-qPCR was carried out as previously described [20]. The change in gene expression due to SIN3 187HA overexpression was compared to wild type S2 cells, while alteration of gene expression due to knockdown of *Sin3A* was compared to GFP RNAi S2 cells. The primers used for RT-qPCR are listed in Additional file 12. The *Taf1* gene was used to normalize the mRNA level. At least three independent biological replicates were performed. Results are represented as the mean of the fold difference between the experimental and control samples.

### RNA-seq and data analysis

Procedures from RNA isolation to next generation sequencing were performed at the Applied Genomics Technology Center, Wayne State University. Briefly, total RNA was extracted using the EZ1® RNA Universal Tissue Kit (Qiagen). Cell lysis and homogenization was carried out by bead-milling on the TissueLyser® II (Qiagen) in 750 μl QIAzol™ lysis reagent. Extracted RNA was purified on the EZ1® Advanced (Qiagen). Additional DNase treatment was done to remove any residual DNA. Quantification and quality assessment of purified total RNA was done using the DropSense96® Microplate Spectrophotometer (Trinean) and microfluidics using the RNA R6K assay for the Agilent 2200 TapeStation, respectively. cDNA was generated by reverse transcription of the purified mRNA. The TruSeq RNA Sample Preparation Kit (Illumina) was used to prepare sequencing libraries following the manufacturer’s protocol. Single indexed samples were multiplexed and sequenced on an Illumina HiSeq 2500 sequencing system in paired end mode with a read length of 2 x 50 bp. A total of three independent biological replicates were performed. In the current study, we report the RNA-seq analysis done on total RNA isolated from cells overexpressing SIN3 187HA. Wild type S2 cells treated in the same way as the SIN3 187HA cell line was used as control. Correlation among replicates is shown in Additional file 13. SIN3 187HA RNA-seq data is available through GEO under GSE72173. The RNA-seq dataset for *Sin3A* knockdown versus GFP RNAi was retrieved through GEO under GSE68775 (Ambikai Gajan, Valerie L. Barnes, Mengying Liu, Nirmalya Saha and Lori A. Pile, unpublished).

The Tophat pipeline was utilized for the analysis [62]. Reads obtained from RNA sequencing were aligned to the UCSC reference genome (dm3) using Bowtie2/Tophat [62]. Cufflinks was used to estimate transcripts levels of a total of 14,540 Refseq genes. Differentially expressed genes were identified using cuffdiff [62] with default parameters at FDR ≤ 0.05 for the *Sin3A* knockdown sample and FDR ≤ 0.001 for the SIN3 187HA overexpression sample. The R statistics environment was used to visualize the data. The volcano and scatter plots were generated using the ggplot2 package.

### GO analysis

Genes bound by SIN3 isoforms and that also showed change in expression upon alteration in the level of SIN3 isoforms were included in the GO analysis. GO analysis, performed separately on genes repressed or activated by SIN3 isoforms, was done utilizing the online tool DAVID [63]. Utilizing visual inspection, we pooled related gene ontology categories into a single broader category. For example, determination of adult lifespan (GO:0008340), multicellular organismal aging (GO:0010259) and aging (GO:0007568) categories found to be enriched for genes activated by SIN3 187, were pooled into one broader category Aging. Detailed information regarding the GO analysis is shown in Additional file 9. GO categories with *P*-value <0.05 were pooled.

### Statistical analysis

Statistical calculations were done using the online statistical tool Graphpad and significant values were calculated using the unpaired Student’s *t*-test.

## Availability of supporting data

ChIP-seq and RNA-seq data can be accessed from the GEO database (www.ncbi.nlm.nih.gov/geo/). ChIP-seq datasets are deposited under accession number GSE72171 and RNA-seq data for SIN3 187 overexpression are deposited under accession number GSE72172. These two datasets are included in a superseries GSE72173. RNA-seq datasets for *Sin3A* knockdown samples can be retrieved from GSE68775.

## Additional files

Additional file 1: Figure S1. ChIP using antibody against SIN3 on chromatin prepared from S2 and SIN3 187HA overexpressing cells (PDF 321 kb) Additional file 2: Figures S2 A, B. The irreproducibility discovery rate analysis showing the correlation between ChIP-seq replicates. This figure is related to Figs. 2 and 3 (PDF 6581 kb) Additional file 3: Table S1. Detailed information on peaks identified by ChIP-seq analysis. This table is related to Figs. 2 and 3 (XLSX 823 kb) Additional file 4: Figure S3 A, B. Venn diagrams showing comparisons between previously published genome-wide SIN3 binding sites to SIN3 isoforms binding events carried out in the current study. This figure is related to Fig. 2 (PDF 590 kb) Additional file 5: Table S2. Detailed annotation of ChIP-seq peaks for SIN3 187HA (SIN3 187HA ceas) or SIN3 220HA (SIN3 220HA ceas) as determined by the *cis*-regulatory enrichment annotation (CEAS) system. This table is related to Fig. 2 (XLSX 4014 kb) Additional file 6: Figure S4. ChIP-seq tracks representing the enrichment of SIN3 isoforms over genes shown in Fig. 1. This figure is related to Fig. 3 (PDF 6815 kb) Additional file 7: Figure S5 A, B. Gene expression changes due to alteration in the levels of SIN3 isoforms. This figure is related to Figs. 4 and 5 (PDF 3258 kb) Additional file 8: Figure S6. ChIP-seq tracks representing enrichment of SIN3 isoforms over *Stat92E*, *Sam-S*, *Gclm* and *tum*. This figure is related to Figs. 4 and 6 (PDF 528 kb) Additional file 9: Table S3. Detailed information of the GO analysis performed on genes directly regulated by SIN3 isoforms. This table is related to Figs. 4 and 6 (XLSX 140 kb) Additional file 10: Table S4. Differential gene expression analysis upon overexpression of SIN3 187HA as determined by Cuffdiff. This table is related Figs. 5 and 6 (XLSX 1669 kb) Additional file 11: Figure S7. RT-qPCR validation of Class D genes (PDF 357 kb) Additional file 12: Table S5. Table listing the primers used in this study (XLSX 10 kb) Additional file 13: Figure S8. Scatter plots representing the correlation between replicates of RNA-seq experiment. This figure is related to Figs. 4, 5 and 6 (PDF 9866 kb)

## Abbreviations

CEAS: *cis*-regulatory enrichment annotation system
ChIP: chromatin immunoprecipitation
ChIP-seq: chromatin immunoprecipitation deep sequencing
dsRNA: double stranded RNA
FPKM: fragments per kilo-base of transcripts per million reads
GO: gene ontology
HDAC: histone deacetylase
IDR: irreproducibility discovery rate
KAT: histone lysine acetyltransferase
KD: knockdown
MNase: micrococcal nuclease
PTMs: post translational modifications
qPCR: quantitative PCR
RNAi: RNA interference
RNA-seq: ribonucleic acid deep sequencing
RT-qPCR: reverse transcriptase and quantitative PCR
TES: transcription end site
TSS: transcription start site
UCSC: University of California, Santa Cruz
UTR: untranslated regions
