# Supplementary material for: Genome-wide studies reveal novel and distinct biological pathways regulated by SIN3 isoforms
Source: BMC Genomics. 2016 Feb 13;17:111. doi: 10.1186/s12864-016-2428-5 (PMC4752761; doi:10.1186/s12864-016-2428-5)
Supplement: Additional file 1: Figure S1. — ChIP using antibody against SIN3 on chromatin prepared from S2 and SIN3 187HA overexpressing cells (PDF 321 kb) [file 12864_2016_2428_MOESM1_ESM.pdf]

Additional file 1

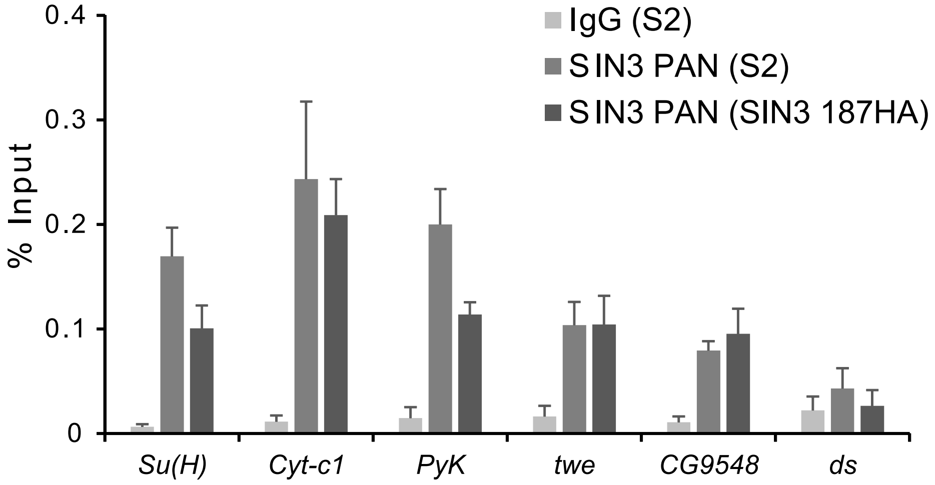

Figure S1. ChIP-qPCR assay performed using antibody against SIN3 on chromatin prepared from S2 cells or cells that were induced to overexpress SIN3 187HA. This figure is related to Figure 1.
