# Supplementary material for: Genome-wide studies reveal novel and distinct biological pathways regulated by SIN3 isoforms
Source: BMC Genomics. 2016 Feb 13;17:111. doi: 10.1186/s12864-016-2428-5 (PMC4752761; doi:10.1186/s12864-016-2428-5)
Supplement: Additional file 2: Figures S2 A, B. — The irreproducibility discovery rate analysis showing the correlation between ChIP-seq replicates. This figure is related to Figs. 2 and 3 (PDF 6581 kb) [file 12864_2016_2428_MOESM2_ESM.pdf]

Additional file 2

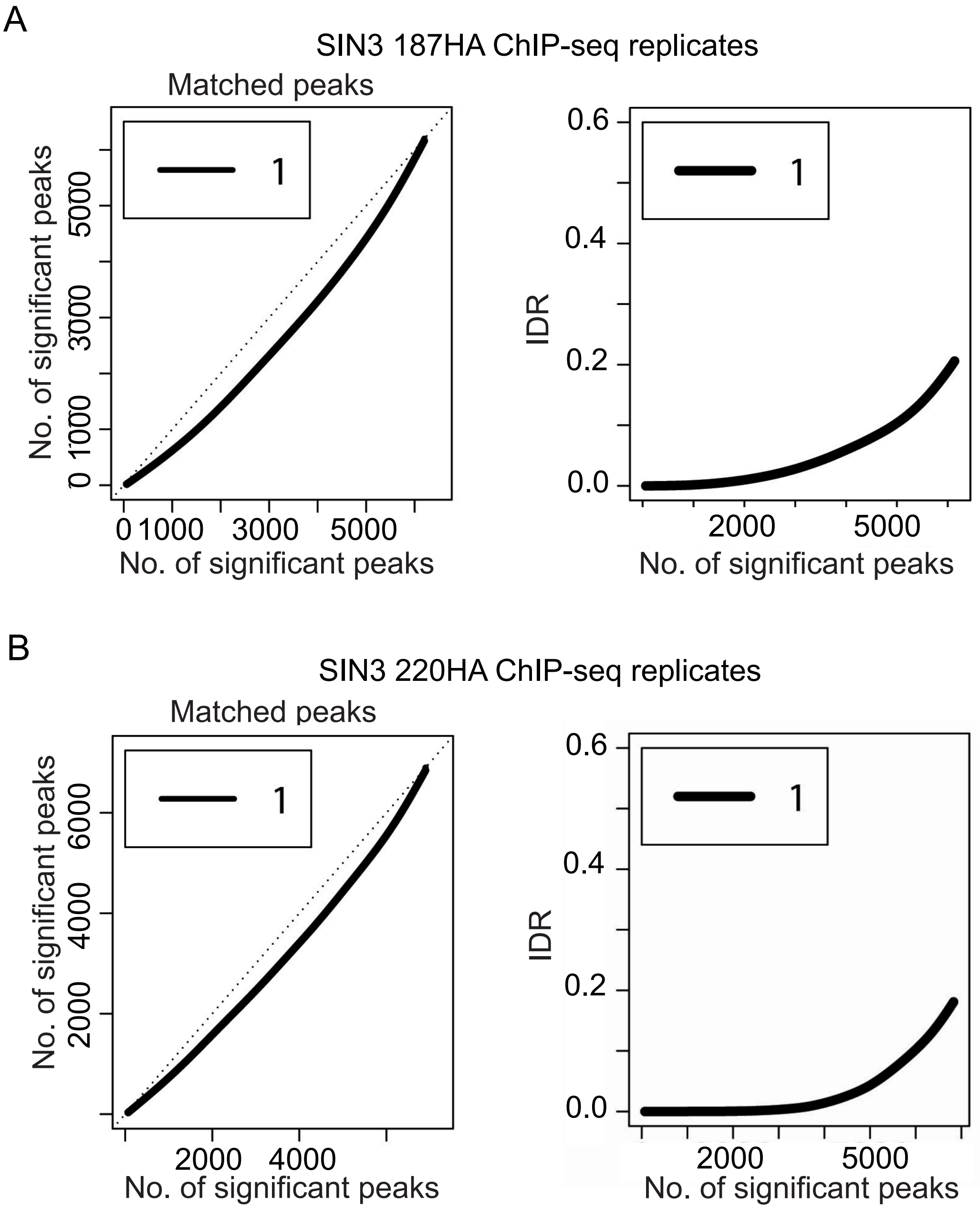

Figure S2. Irreproducible discovery rate (IDR) analysis showing the correlation between the two biological replicates of ChIP-seq analysis for SIN3 187HA samples (A) and SIN3 220HA samples (B). This figure is related to Figure 2, 3.
