# Supplementary material for: Genome-wide studies reveal novel and distinct biological pathways regulated by SIN3 isoforms
Source: BMC Genomics. 2016 Feb 13;17:111. doi: 10.1186/s12864-016-2428-5 (PMC4752761; doi:10.1186/s12864-016-2428-5)
Supplement: Additional file 4: Figure S3 A, B. — Venn diagrams showing comparisons between previously published genome-wide SIN3 binding sites to SIN3 isoforms binding events carried out in the current study. This figure is related to Fig. 2 (PDF 590 kb) [file 12864_2016_2428_MOESM4_ESM.pdf]

Additional file 4

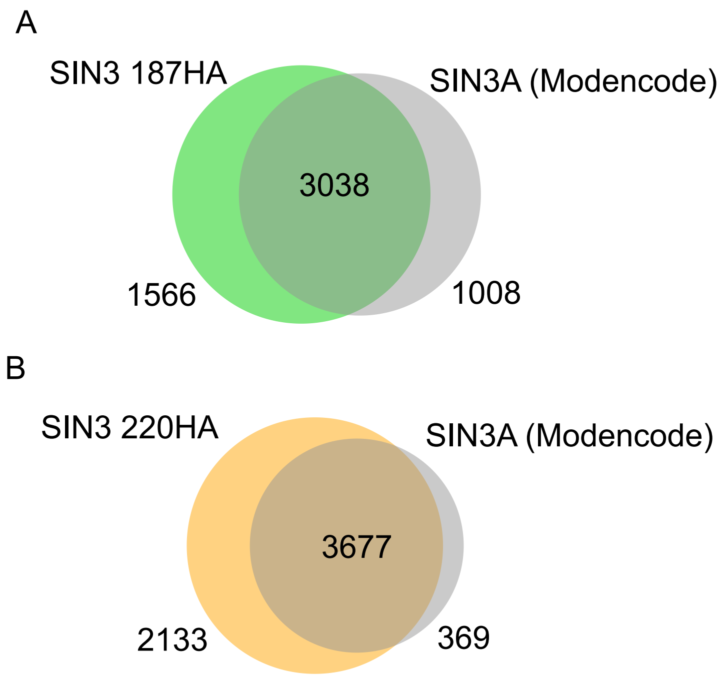

Figure S3. Venn diagrams showing the comparison of the number of peaks identified in this study for SIN3 187HA (A) or SIN3 220HA (B) to the number of peaks identified previously (Modencode) (Negre, Brown et al. 2011). This figure is related to Figure 2.
