# Supplementary material for: Genome-wide studies reveal novel and distinct biological pathways regulated by SIN3 isoforms
Source: BMC Genomics. 2016 Feb 13;17:111. doi: 10.1186/s12864-016-2428-5 (PMC4752761; doi:10.1186/s12864-016-2428-5)
Supplement: Additional file 6: Figure S4. — ChIP-seq tracks representing the enrichment of SIN3 isoforms over genes shown in Fig. 1. This figure is related to Fig. 3 (PDF 6815 kb) [file 12864_2016_2428_MOESM6_ESM.pdf]

Additional file 6

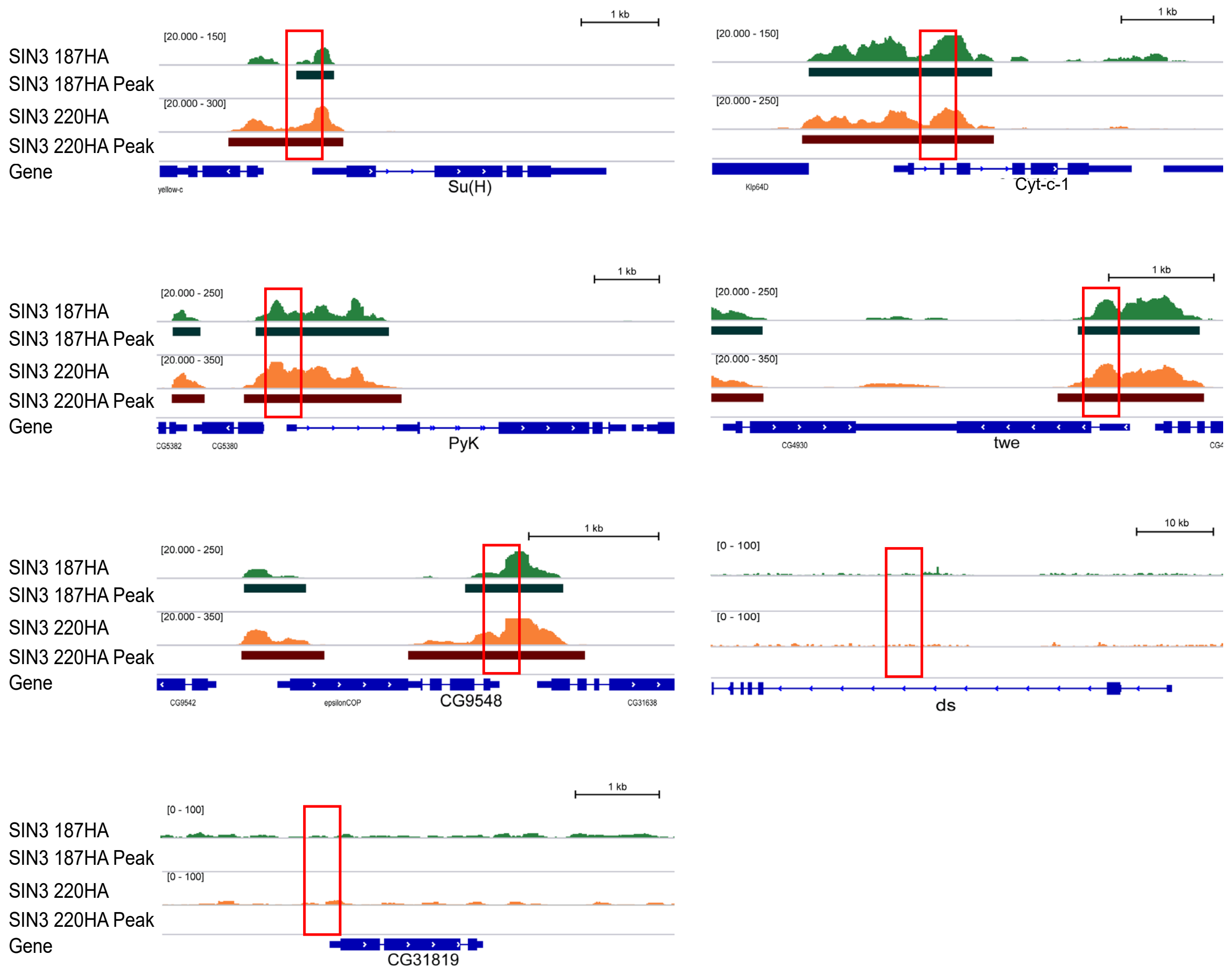

Figure S4. ChIP-seq gene tracks showing the enrichment of SIN3 isoforms over the genes shown in Figure 1C and D. Red boxes mark the approximate region amplified by qPCR following ChIP as shown in Figure 1C and D. This figure is related to Figure 3A.
