# Supplementary material for: Genome-wide studies reveal novel and distinct biological pathways regulated by SIN3 isoforms
Source: BMC Genomics. 2016 Feb 13;17:111. doi: 10.1186/s12864-016-2428-5 (PMC4752761; doi:10.1186/s12864-016-2428-5)
Supplement: Additional file 7: Figure S5 A, B. — Gene expression changes due to alteration in the levels of SIN3 isoforms. This figure is related to Figs. 4 and 5 (PDF 3258 kb) [file 12864_2016_2428_MOESM7_ESM.pdf]

Additional file 7

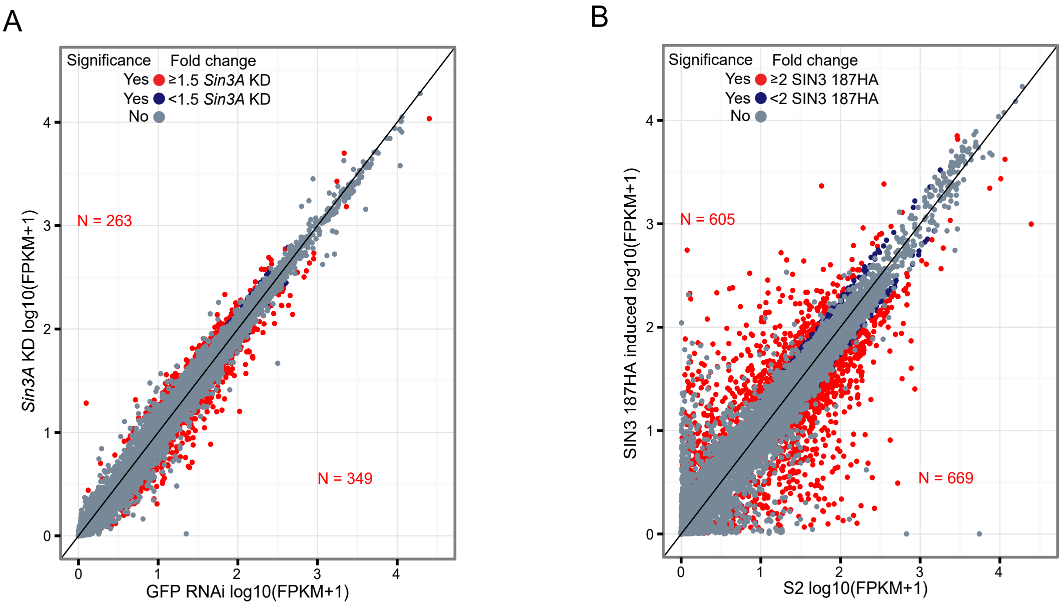

Figure S5. Scatter plots showing the gene expression change due to *Sin3A* knockdown (KD) compared to GFP RNAi (A) and SIN3 187HA overexpression versus S2 mock induced cells (B).
