# Supplementary material for: Genome-wide studies reveal novel and distinct biological pathways regulated by SIN3 isoforms
Source: BMC Genomics. 2016 Feb 13;17:111. doi: 10.1186/s12864-016-2428-5 (PMC4752761; doi:10.1186/s12864-016-2428-5)
Supplement: Additional file 8: Figure S6. — ChIP-seq tracks representing enrichment of SIN3 isoforms over Stat92E, Sam-S, Gclm and tum. This figure is related to Figs. 4 and 6 (PDF 528 kb) [file 12864_2016_2428_MOESM8_ESM.pdf]

Additional file 8

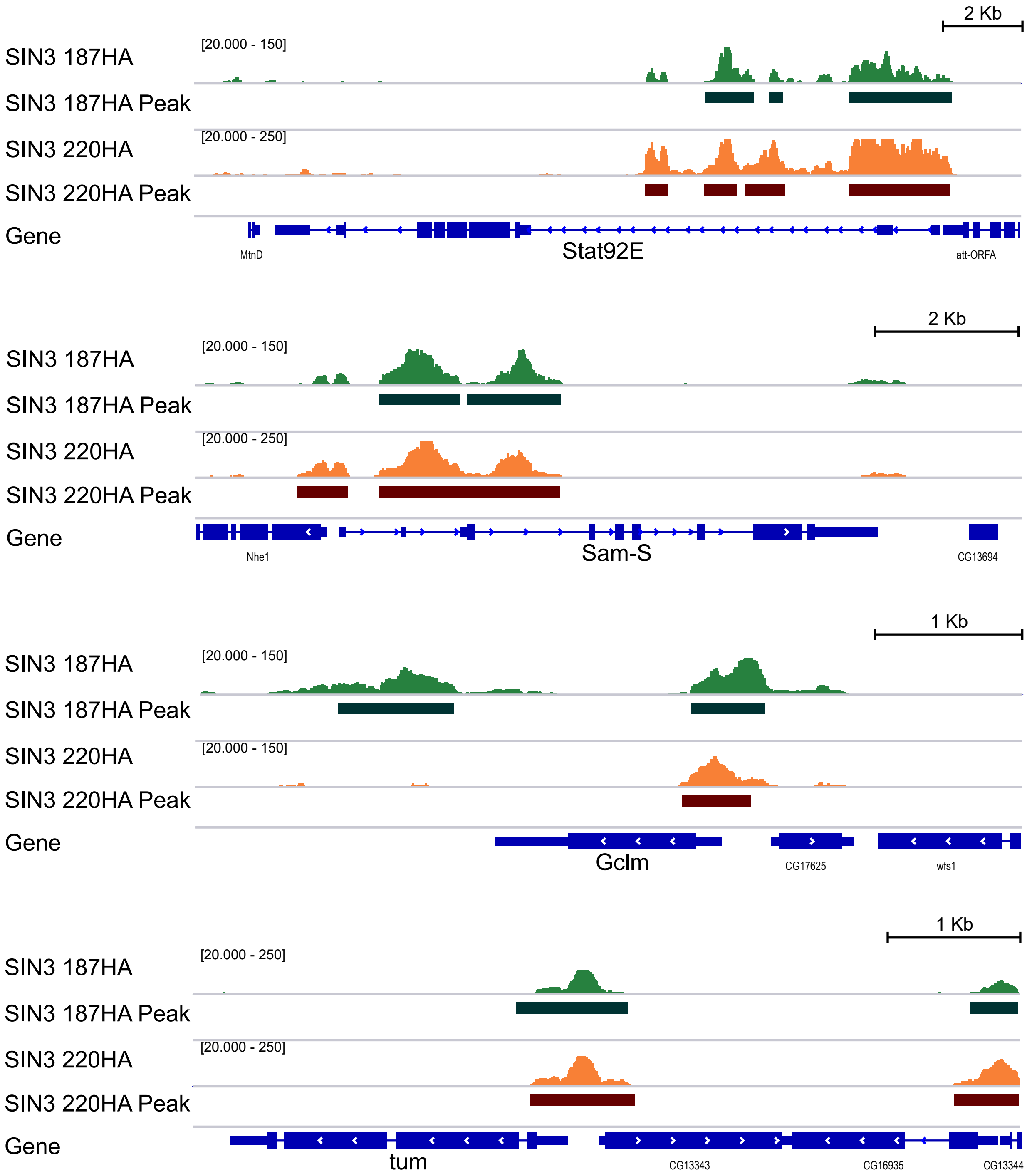

Figure S6. ChIP-seq gene tracks showing enrichment of SIN3 isoforms over *Stat92E*, *Sam-S*, *Gclm* and *tum*.
