# Supplementary material for: Genome-wide studies reveal novel and distinct biological pathways regulated by SIN3 isoforms
Source: BMC Genomics. 2016 Feb 13;17:111. doi: 10.1186/s12864-016-2428-5 (PMC4752761; doi:10.1186/s12864-016-2428-5)
Supplement: Additional file 11: Figure S7. — RT-qPCR validation of Class D genes (PDF 357 kb) [file 12864_2016_2428_MOESM11_ESM.pdf]

Additional file 11

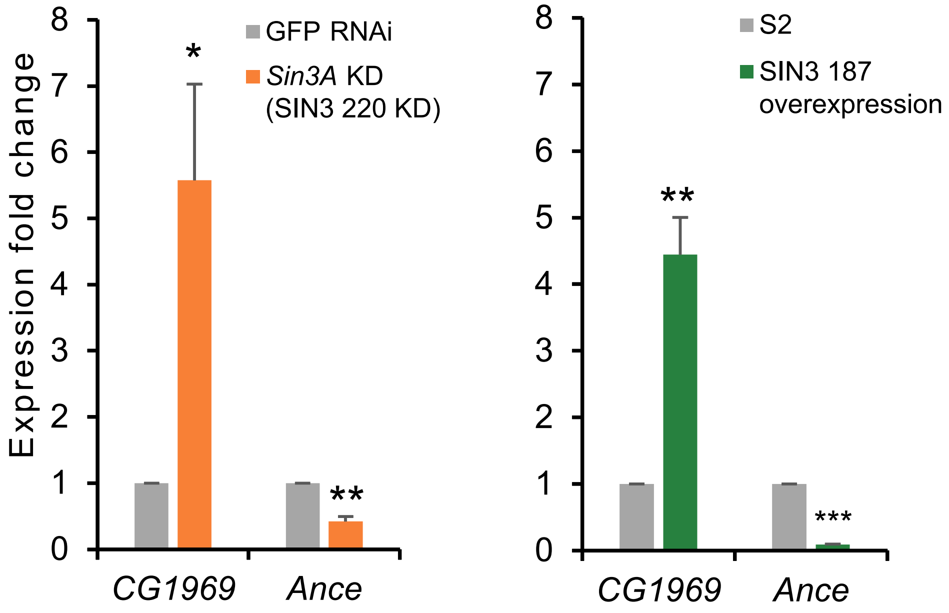

Figure S7. RT-qPCR verification of Class D genes. The level of *CG1969* mRNA is upregulated upon *Sin3A* (SIN3 220) knockdown or SIN3 187 overexpression indicating that this gene is repressed by SIN3 220 and activated by SIN3 187. Conversely, expression of *Ance* is downregulated due to alteration in the level of SIN3 isoforms demonstrating that SIN3 220 and SIN3 187 act as a co-activator or as a co-repressor, respectively.

This figure is related to Figure 5. *P*-value * < 0.05, ** < 0.01, *** < 0.0001.
