# Supplementary material for: Genome-wide studies reveal novel and distinct biological pathways regulated by SIN3 isoforms
Source: BMC Genomics. 2016 Feb 13;17:111. doi: 10.1186/s12864-016-2428-5 (PMC4752761; doi:10.1186/s12864-016-2428-5)
Supplement: Additional file 13: Figure S8. — Scatter plots representing the correlation between replicates of RNA-seq experiment. This figure is related to Figs. 4, 5 and 6 (PDF 9866 kb) [file 12864_2016_2428_MOESM13_ESM.pdf]

Additional file 13

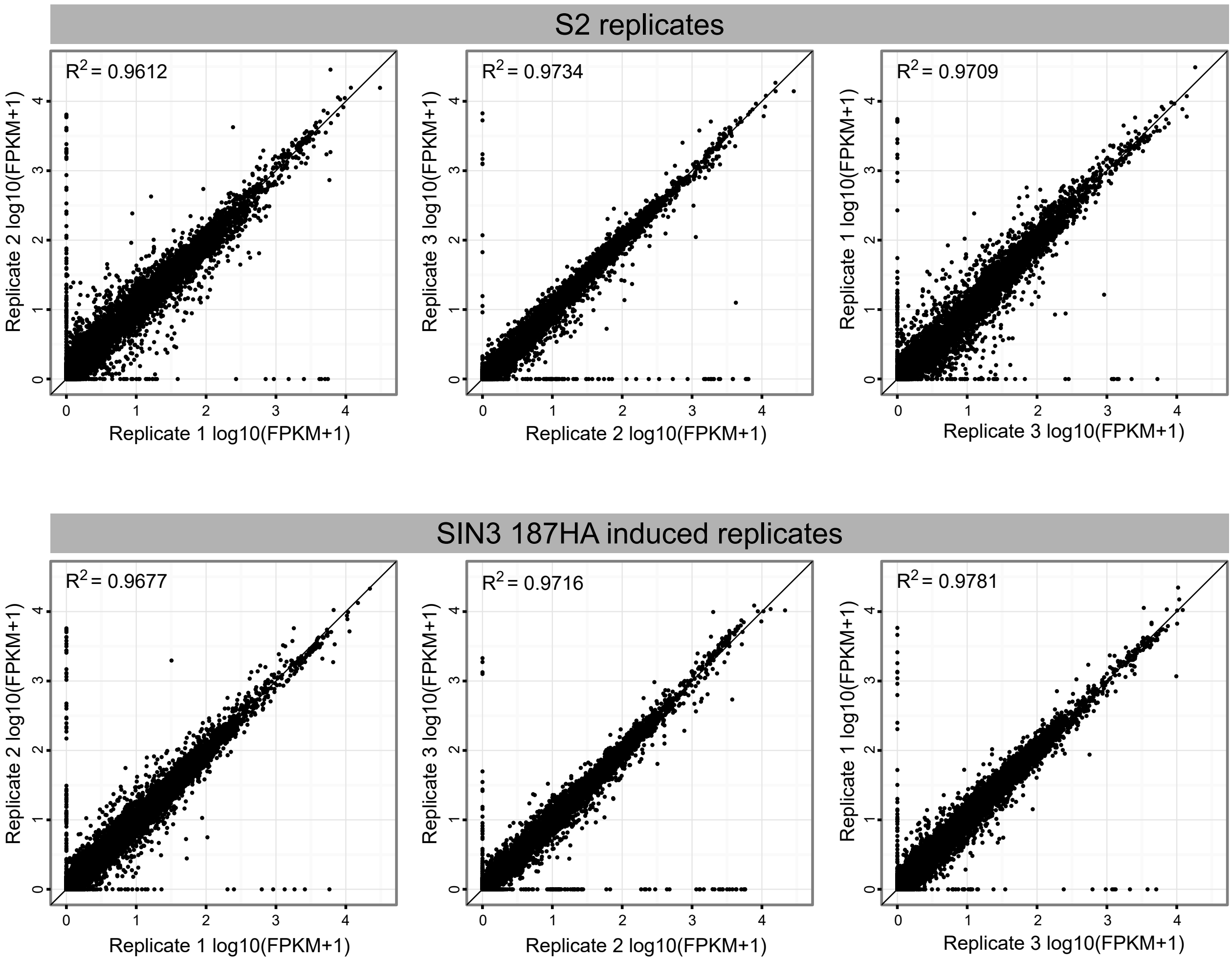

Figure S8. Scatter plots showing the correlation between the replicates of gene expression analysis by RNA-seq using total RNA extracted from S2 cells (top) or the SIN3 187HA stably transfected cell line (bottom). Data representing the correlation between three independent biological replicates. This figure is related to Figures 5 and 6.
